# Investigating the Anticancer Activity of G-Rh1 Using In Silico and In Vitro Studies (A549 Lung Cancer Cells)

**DOI:** 10.3390/molecules27238311

**Published:** 2022-11-28

**Authors:** Jinnatun Nahar, Vinothini Boopathi, Mohanapriya Murugesan, Esrat Jahan Rupa, Deok Chun Yang, Se Chan Kang, Ramya Mathiyalagan

**Affiliations:** 1 Graduate School of Biotechnology, College of Life Sciences, Kyung Hee University, Yongin si 17104, Gyeonggi do, Republic of Korea; jinnatunnaharbph@gmail.com (J.N.); vinothini9327@gmail.com (V.B.); priyabuddy44@gmail.com (M.M.); dcyang@khu.ac.kr (D.C.Y.); 2 Department of Oriental Medicinal Biotechnology, College of Life Science, Kyung Hee University, Yongin-si 17104, Gyeonggi-do, Republic of Korea; eshratrupa91@gmail.com

**Keywords:** ROCK1, ADMET, Insilco, autodock vina, anti-lung cancer

## Abstract

Ginsenoside Rh1 (G-Rh1), a possible bioactive substance isolated from the Korean *Panax ginseng* Meyer, has a wide range of pharmacological effects. In this study, we have investigated the anticancer efficacy of G-Rh1 via in silico and in vitro methodologies. This study mainly focuses on the two metastatic regulators, Rho-associated protein kinase 1 (ROCK1) and RhoA, along with other standard apoptosis regulators. The ROCK1 protein is a member of the active serine/threonine kinase family that is crucial for many biological processes, including cell division, differentiation, and death, as well as many cellular processes and muscle contraction. The abnormal activation of ROCK1 kinase causes several disorders, whereas numerous studies have also shown that RhoA is expressed highly in various cancers, including colon, lung, ovarian, gastric, and liver malignancies. Hence, inhibiting both ROCK1 and RhoA will be promising in preventing metastasis. Therefore, the molecular level interaction of G-Rh1 with the ROCK1 and RhoA active site residues from the preliminary screening clearly shows its inhibitory potential. Molecular dynamics simulation and principal component analysis give essential insights for comprehending the conformational changes that result from G-Rh1 binding to ROCK1 and RhoA. Further, MTT assay was employed to examine the potential cytotoxicity in vitro against human lung cancer cells (A549) and Raw 264.7 Murine macrophage cells. Thus, G-Rh1 showed significant cytotoxicity against human lung adenocarcinoma (A549) at 100 µg/mL. In addition, we observed an elevated level of reactive oxygen species (ROS) generation, perhaps promoting cancer cell toxicity. Additionally, G-Rh1 suppressed the mRNA expression of RhoA, ROCK1, MMP1, and MMP9 in cancer cell. Accordingly, G-Rh1 upregulated the p53, Bax, Caspase 3, caspase 9 while Bcl2 is downregulated intrinsic pathway. The findings from our study propose that the anticancer activity of G-Rh1 may be related to the induction of apoptosis by the RhoA/ROCK1 signaling pathway. As a result, this study evaluated the functional drug-like compound G-Rh1 from *Panax ginseng* in preventing and treating lung cancer adenocarcinoma via regulating metastasis and apoptosis.

## 1. Introduction

According to Global Cancer Statistics 2020, cancer is a terrible life-threatening disease globally, with a higher mortality rate of approximately 19.3 million new cancer cases and almost 10·0 million deaths. Among various cancers, lung cancer remained the leading cause of cancer-related death, with an estimated 1.8 million deaths and 2.20 million new cases per year [1]. Non-small cell lung cancer (NSCLC) is primarily accountable for lung malignancy, accounting for 85% of all lung cancers with lower therapeutic activity [2]. Today, various conventional medications such as chemotherapies, radiation, and surgery are used in treating cancer. Although combining different therapy increases cancer patients’ chances of prolonged survival, the ultimate result is still unsatisfactory because of adverse effects with drug resistance [3,4,5]. Therefore, there is an urgent need to extend novel therapeutic approaches to improve lung cancer patients’ survival with fewer or no side effects. In this critical situation, natural products play a significant role in cancer prevention and treatment because they have unique efficacy, safety, and economic impact on cancer [6,7].

**Figure 1:**
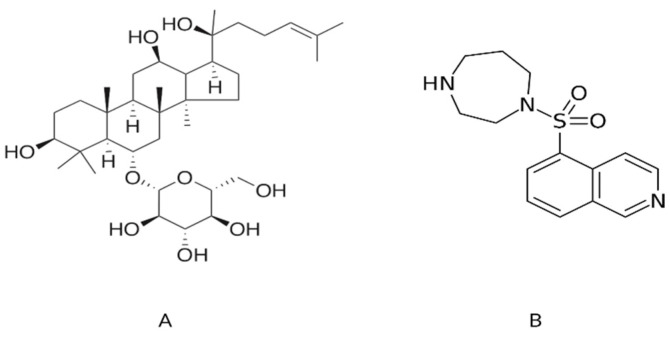
The two-dimensional graphical representation of (**A**) ginsenoside Rh1 from *Panax ginseng* and (**B**) fasudil, a known inhibitor of ROCK1.

The hallmarks of cancer comprise six biological capabilities acquired during the multistep development of human tumors. They include sustaining proliferative signaling, evading growth suppressors, resisting cell death, enabling replicative immortality, inducing angiogenesis, and activating invasion and metastasis [8]. Metastasis is a multifactorial and multicellular process involving actin structures’ dynamic formation and breakdown [9]. The ROCK protein has two isomers, ROCK1 and ROCK2, and both display 92% homology in the catalytic kinase domain and 65% identity in their overall amino acid sequences [10,11]. The human chromosomes 18 (18q11.1) and 2 (2p24) contain the ROCK1 and ROCK2 genes, respectively [12]. Additionally, the primary upstream regulator of ROCK is the Rho protein, which belongs to the Ras superfamily and is a small-molecule GTP-binding protein with low GTP enzyme activity. By interacting with GTP or GDP conformation, it can alternate between high and low activity [13]. Additionally, numerous studies have shown that abnormal activation of ROCK kinase leads to a variety of diseases such as cancer [9,14], cardiovascular disorders [15], nervous system diseases [16], hypertension [17], Alzheimer’s disease [18], myocardial ischemia [19], heart failure and kidney failure [20]. Consequently, several potential ROCK inhibitors are currently in clinical trials, and only one compound, fasudil (Figure 1B), has been approved as a ROCK inhibitor (https://clinicaltrials.gov/ct2/show/NCT03792490 (accessed on 10 October 2022)) [21]. Fasudil has been marketed in Japan since 1995 and is used for treating cerebral vasospasm and ischemia [22]. ROCK1 protein is considered an important drug target for several diseases, so it is critical to identify additional potential ROCK1 inhibitors.

A widely expressed, highly conserved serine/threonine kinase with a molecular weight of approximately 160 kDa called ROCK (also known as ROCK1 and ROCK2) is a crucial downstream effector controlled by the small GTPase RhoA [23]. Rho GTPases, which are members of the Ras GTPase superfamily and share 25% homology with Ras, were first identified in 1985 [21]. One of the essential Rho GTPase members is RhoA/B/C. Hence, it is another significant protein family controlling cell migration is Rho GTPase, which is essential for maintaining cell morphology, motility, and cell-cell and cell-matrix adhesion RhoA is a small GTPase protein belonging to the Rho family and is connected to regulating the actin cytoskeleton [22]. The control of cell motility in the actin cytoskeleton creates the potential for regulating tumor cell metastasis [24]. Therefore, Rho GTPases bind to many effector proteins and play central roles in regulating the actin and microtubule cytoskeletons and gene transcription [25]. These proteins have been implicated in many critical cancer-related processes in mammalian cells, such as proliferation, migration, and survival. Moreover, RhoA/ROCK signaling plays a crucial role in various human diseases. It is now considered a potential target for the treatment of several diseases, including lung cancer [26,27], breast cancer [28] gastric cancer [29], and colon cancer [30]. Therefore, RhoA/ROCK1 could be an ideal candidate target in lung cancer treatment as it is found to be highly expressed in cancer. Hence, inhibiting or suppressing these two targets will be a promising strategy in preventing and managing cancer.

On the other hand, reactive oxygen species (ROS) are extracellular mediators that support various signaling pathways, including cancer metastasis and proliferation [31]. Reactive oxygen species (ROS) overproduction is a hallmark of mitochondria in cancer cells that aids in the progression of the disease by causing genomic instability, altering gene expression, and actively participating in signaling pathways [32]. Mitochondrial ROS regulates mitochondrial mechanisms involved in cancer homeostasis and development [33]. Previous reports demonstrated that ROS could regulate cancer proliferation and apoptosis by the signaling pathway of p53/bax/bcl2 [34,35,36]. Additionally, MMPs comprise a structurally and functionally related family of zinc metalloproteinases degrading extracellular matrix and basement membrane barriers and thus are thought to play a key role in angiogenesis, inflammatory processes, cancer development, cell proliferation, and apoptosis [37]. A recent study revealed that in hepatocellular carcinoma, MMP-1 is a poor prognostic biomarker for patients [38]. In addition, the impact of MMP-1 and MMP-9 levels, and consequently the prevalence and development of breast cancer, on 1G/2G and CT polymorphisms [39]. Therefore, the above-mentioned targets play crucial role in apoptosis, hence regulating them will be favorable in the treatment of lung cancer.

*Panax ginseng*, commonly known as ginseng, is an attractive natural medicinal plant used worldwide in East Asian countries, including Korea, China, and Japan [40]. The major components of ginseng are ginsenosides, which contain an aglycone with a dammarane skeleton [41]. Ginsenosides appear to be responsible for most of the activities of ginseng, including the anti-inflammatory, anti-apoptosis, cardiovascular diseases, and other effects [42,43]. Additionally, Red ginseng contains ginsenoside Rh1, a metabolite of the significant ginsenosides Re and Rg1, whereas Rh1 is created by intestinal microbiota after oral use of ginseng [44,45]. Moreover, the mechanisms and anticancer effects of *P. ginseng* and its metabolites (CK, G-Rh1, Rh2, Rh3, and F1) in various cancers (breast cancer, colon cancer, prostate cancer, stomach cancer, and lung cancer) have been discussed in several studies [46,47]. Among all the *P. ginseng* saponins, G-Rh1 is a potential bioactive compound identified from roots, leaves, stems, fruits, and flower buds [20]. In addition, G-Rh1 induces anticancer activities in several cancer cells, including Breast cancer [48], colorectal cancer cell [47], human hepatocellular carcinoma [49], astroglioma [50] and acute monocytic leukemia cells [51].

The requirement to determine the molecular interactions between G-Rh1 and bio-macromolecules is crucial in developing natural products as drugs. Computer-aided drug design (CADD) effectively uses in silico methodologies such as molecular docking, molecular dynamics, pharmacophore modeling, and chemoinformatics tools to improve and refine therapeutic candidates derived from natural sources [52,53]. This study used comprehensive simulations to assess the structural stability, conformational changes, and protein movements of the ginsenoside Rh1-RhoA and Rh1-ROCK1 complexes. The G-Rh1 chemical space interacted with the RhoA and ROCK1 binding active sites with a suitable binding mode. This was incorporated into the molecular dynamics study and principal component analysis. Although several studies have investigated the anticancer effects of Rh1, the underlying mechanisms of Rh1 on lung cancer migration and invasion remain unknown. Finally, in the present study, we investigated the regulation of lung cancer invasion and migration by G-Rh1 with RhoA/Rock/p53/MMP-1/MMP-9 pathway through in vitro evaluation following the in silico confirmation.

## 2. Results and Discussion

### 2.1. Pharmacokinetic Properties of G-Rh1

The 2D structure of G-Rh1 from *Panax ginseng* was drawn, and energy was minimized (Figure 1). The prepared ginsenoside Rh1 structure was used to generate the molecule’s physiochemical and pharmacokinetic properties. We determined various calculated properties such as aqueous solubility (accepted range: 6.5 to −0.5), serum protein binding (accepted range: 1.5 to −1.5), logP for octanol/water (accepted range: −0.2 to 6.5), hepatotoxicity (accepted range: nontoxic is 0 and toxic is 1), CYP2D6 inhibition probability (accepted range: noninhibitor is 0 and inhibitor is 1), and human oral absorption in the gastrointestinal tract (accepted range: poor is <25% and high is >80%). The detailed pharmacokinetic (ADMET) results of G-Rh1 are shown in Table 1. The ADMET descriptors for ginsenoside Rh1 are as follows: retrieved aqueous solubility (−4.829), serum protein binding (0.1), logP for octanol/water (2.6), hepatotoxicity (0), CYP2D6 inhibition probability (0.2), and human oral absorption in GI (49.4%). The predicted G-Rh1 values fall in the accepted ranges of 95% of known drugs. These results clearly indicate that ginsenoside Rh1 is an orally bio-active compound and can be used for targeting the disease.

The drug-likeness filtration study was performed for G-Rh1 based on Lipinski rules. The drug-like molecules were predicted according to the following rules: MW (<500), LogP (<5), hydrogen bond donor (HBD) (<5), and hydrogen bond acceptor (HBA) (<10). G-Rh1 shows a molecular weight of 638 kDa, but this is in the accepted range of 95% of known drugs. HBD [7] and HBA [17] are Lipinski rule violations, but they were considered to be the discovery of drug molecules from natural products [55]. Then, LogP of 2.6 also conformed to drug-like properties, and the predicted descriptors of ginsenoside Rh1 are shown in in Table 2.

PASS, which predicts the biological activity spectrum based on the chemical structure formula, showed significant biological targets for G-Rh1. This program was applied to the multilevel neighborhoods of atoms (MNA) descriptor and was used to calculate the targets from the original chemical structure. These MNA descriptors can be used to predict the Pa and Pi scores, which range from 0 to 1 from G-Rh1. The biological target active molecules can be identified based on Pa values close to 1 and Pi close to 0 [56]. The results of G-Rh1 showed probable biological activity to the following targets: chemo preventive, caspase 3 stimulant, dementia treatment, vascular dementia treatment, antithrombotic, CYP3A inducer, CYP3A4 inducer, apoptosis antagonist, hepatoprotection, and CYP2C9 inducer. The predicted targets for G-Rh1 along with the Pa and Pi values are shown in Table 3.

### 2.2. Molecular Interaction Results of ROCK1 and RhoA with Ginsenoside Rh1

The molecular-level interaction of ROCK1 protein and RhoA with ginsenoside Rh1 was analyzed using the molecular docking method. One gets the impression that docking was especially designed for natural compounds. It is a method that predicts the most energetically favorable orientation of a ligand (usually a small molecule but could also be a biopolymer) to a receptor (usually a protein). In this study, the ROCK1 and RhoA crystal structure was used to perform the docking simulation using the Autodock Vina program. The essential active site residues were kept flexible, and fasudil and dexamethasone was used as a control. The interaction results were confirmed by their hydrogen bond formation and binding energy to the crucial active residues and ginsenoside Rh1. Analysis of docking results shows that ginsenoside Rh1 interacts with ROCK1 via four hydrogen bonds (ALA86, ASP160, ASN203, ASP216) to ROCK1 active site residues along with −8.9 kcal/mol binding affinities. Three hydrophobic interactions with amino acid residues GLY85, ILE82, VAL162 has been formed. Interestingly G-Rh1 shares two similar H-bond interaction as the control drugs used.

**Figure 2:**
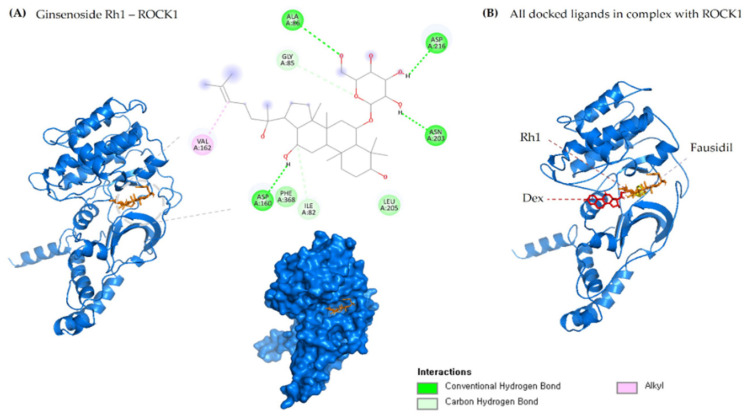
(**A**) 2D & 3D docking interactions of Ginsenoside Rh1 with ROCK1. (**B**) All docked ligands in complex with ROCK1 (Orange—Rh1, Pink—Dexamethasone, Yellow—Fausidil).

Analysis of docking results shows that ginsenoside Rh1 interacts with RhoA1 via four hydrogen bonds (ARG5, ASP78, PRO180, GLN180) to RhoA active site residues along with −7.1 kcal/mol binding affinities. Further, G-Rh1 forms four hydrophobic interactions with the amino acid residues ASP78, LYS6, PRO75, PHE106. It also binds to the similar binding pocket as dexamethasone and GDP. The detailed docking results along with the control ligand are shown in Table 4. A graphical representation of ginsenoside Rh1 interacting with the ROCK1 active site residues is shown in Figure 2A. A graphical representation of ginsenoside Rh1 interacting with the ROCK1 active site residues is shown in Figure 2B. Other reports have shown that these two strongest hydrogen bonds are implicated in ROCK1 inhibition in numerous diseases and have been used for targeted development of potential drug candidates [57,58].

### 2.3. Molecular Dynamics Simulation, MM-PBSA and PCA Analysis

**Figure 3:**
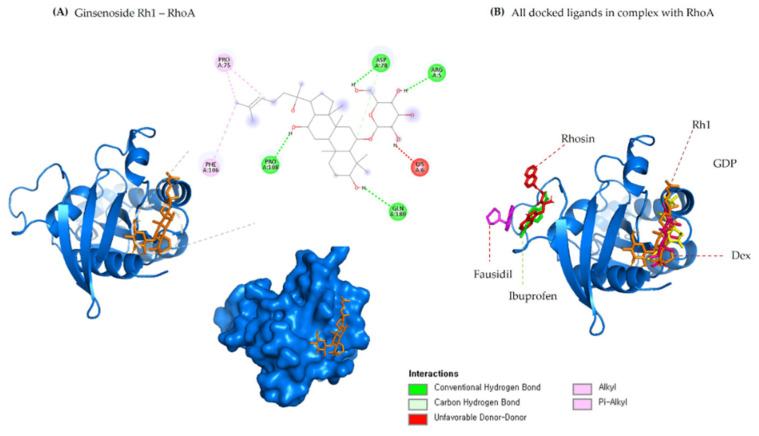
(**A**) 2D & 3D docking interactions of Ginsenoside Rh1 with RhoA. (**B)** All docked ligands in complex with RhoA (Orange—Rh1, Pink—Dexamethasone, Yellow—GDP, Magenta—Fausidil, Red—Rhosin, Green—Ibuprofen).

**Figure 4:**
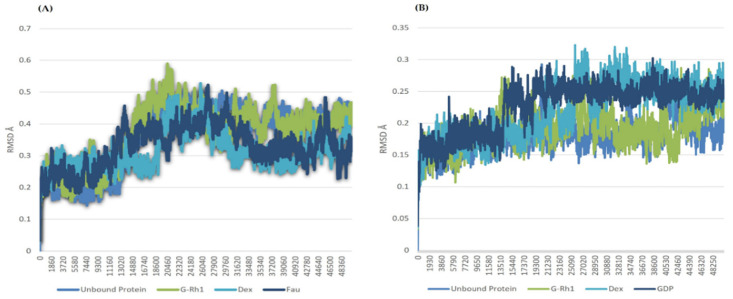
RMSD of ginsenosides Rh1 and control (Dexamethasone, Fausidil) in complex with (**A**) ROCK1 and (**B**) G-Rh1 and control (Dexamethasone, GDP) in complex with RhoA as a function of MD simulation time.

**Figure 5:**
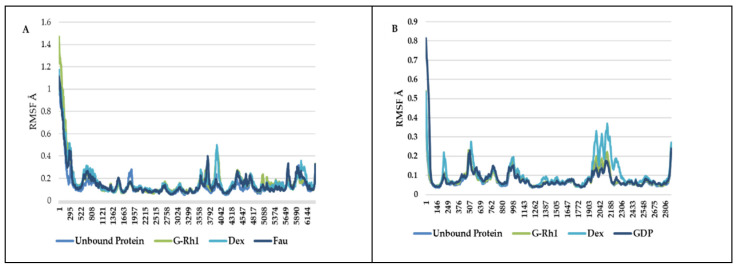
RMSF of ginsenosides Rh1 and control (Dexamethasone, Fausidil) in complex with (**A**) ROCK1 and (**B**) G-Rh1 and control (Dexamethasone, GDP) in complex with RhoA as a function of MD simulation time.

**Figure 6:**
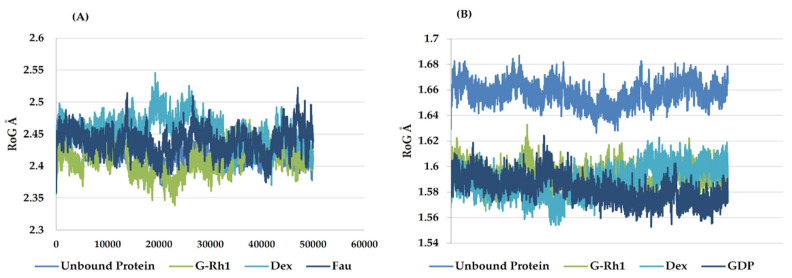
Radius of gyration plots of molecular dynamics (MD) simulation of (**A**) ROCK1 receptor-ginsenoside Rh1 and control (Dexamethasone, Fausidil) complexes and (**B**) RhoA receptor-ginsenoside Rh1 and control (Dexamethasone, GDP) complexes.

**Figure 7:**
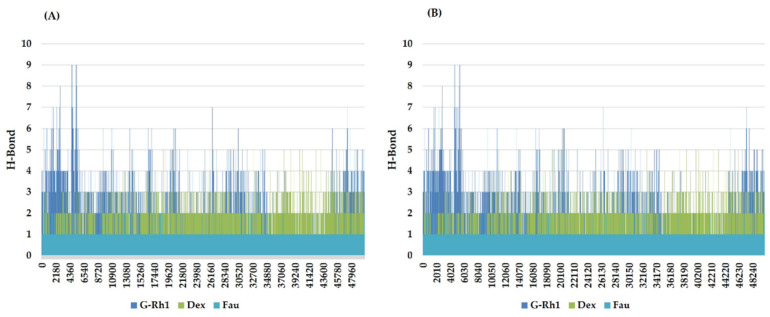
Line plots of Ligand-protein H bonds for (**A**) ROCK1 and (**B**) RhoA with Ginsenosides Rh1 and control (Dexamethasone, Fausidil).

The structural evolution was employed to the docked complex structures of ROCK1 (Figure S2), and RhoA (Figure S3) interaction using the molecular dynamics (MD) method. In this study, three individual MD simulations were performed on the apo form of ROCK1 and RhoA along with the complexes of ROCK1-fasudilROCK1-dexamethasone, ROCK1-ginsenoside Rh1, RhoA—ginsenoside Rh1, RhoA—dexamethasone, and RhoA—GDP (Figure 3A,B). Analysis of MD results (Figure 4) show the root mean square deviation (RMSD) against the backbone of each complex with an attained equilibration around 1.5 ns (ROCK1-fasudil), and 0.7 ns (ROCK1-ginsenoside Rh1 and RhoA—ginsenoside Rh1). After obtaining the equilibration of each complex, stability was maintained throughout the entire 50 ns simulation time. In addition, root mean square fluctuation (RMSF) analysis of the ROCK1-ginsenoside Rh1 complex and RhoA—ginsenoside Rh1 were calculated throughout the entire simulation against Cα atoms. For the analysis of trajectory files, important active site residues of ROCK1 and RhoA with ginsenoside Rh1 were compared with the ROCK1-fasudil complex and RhoA -fasudil complex a known inhibitor. Figure 5 displays the obtained RMSF values of each complex. The radius of gyration and H-bond analysis also confirmed the docking result (Figure 6 and Figure 7).

**Figure 8:**
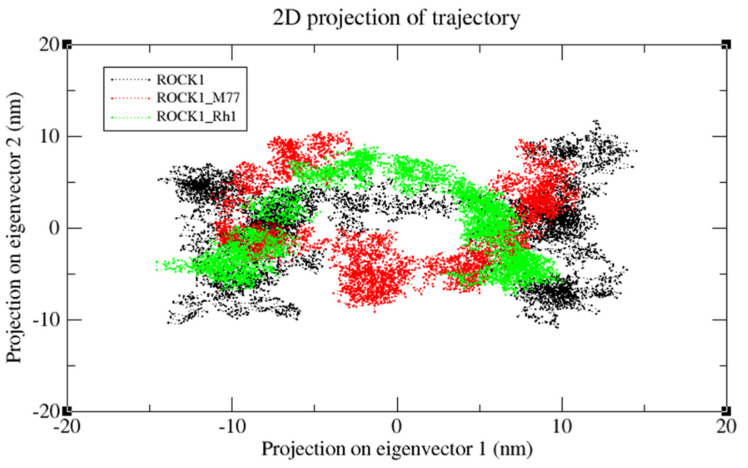
The cloud represents a 10 ns trajectory projected with the first two eigenvectors. PCA re-sults of ROCK1 protein, ROCK1-fasudil, and ROCK1-ginsenosde Rh1 trajectories are shown in black, red, and green, respectively.

We also applied the DSSP algorithm [59] to examine the secondary structural changes in ROCK1 protein and ROCK1-ginsenoside complex during the entire simulation. The observed differences in secondary structural elements are shown in Figure S1. The binding affinity scores were computed before and after dynamic snapshots (Table 5) for the ROCK1-ginsenoside Rh1 complex structure. For the detailed analysis, different time scale snapshots were used to determine the conformational changes upon ginsenoside Rh1 binding to the ROCK1 protein. For this analysis, every 2 ns snapshot was collected from the entire 10 ns trajectory and different time scales were analyzed using the VMD program (Figure S4). We also used this 10 ns trajectory to perform PCA to examine protein motions with principal components. The backbone atoms of each structure in the PCA spectrum indicate the level of atomic fluctuations and behavior of protein motions based on the first two eigenvectors. Both ROCK1 protein and ROCK1-ginsenoside Rh1 complex structures cover a small space in that plane of 2D projection. The cloud represents the 10 ns trajectory projected with the first two eigenvectors and is shown in Figure 8. From the MD results, the backbone RMSD, residue level fluctuations of Cα atoms, secondary structure conformational changes, and protein motions based on principal component values clearly show the molecular mechanism of ROCK1 and RhoA upon binding to ginsenoside Rh1. Finally, we performed the MM-PBSA analysis to calculate the thermodynamics parameters of the complex, such as binding free/van der Waals/electrostatic/polar solvation energies (ΔE_binding_; E_vdw_; E_elec_; ΔE_polar_ respectively) at the molecular level. Table 5 displays the calculation result, indicating that each complex’s BFE value is greater than the control complex value.

### 2.4. Cytotoxicity Effect of Ginsenosides Rh1

**Figure 9:**
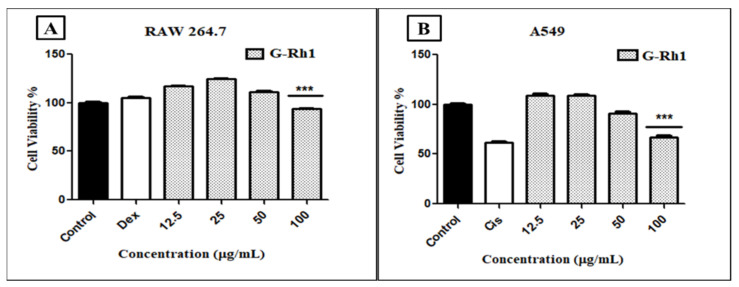
In vitro, cell cytotoxicity evaluation for ginsenosides Rh1 (**A**) on murine macrophage (RAW 264.7), (**B**) on human lg carcinoma (A549) cells compared to positive control (cisplatin). Graph shows mean ± SD values of four replicates. *** *p* < 0.001 indicates significant differences from control groups.

Natural bioactive chemicals are potential sources for developing drugs to treat various diseases. In particular, several ginsenosides have been shown to have pharmacological effects, including anticancer properties [51,55,60]. Among them, Rh1 has been shown to suppress a variety of cancer cell lines, including breast cancer cells, lung cancer A549 cells, and cervical cancer HeLa cells [61]. Our findings observed the cytotoxicity level of the G- Rh1 on murine macrophage (RAW 264.7) cells, and A549 lung cancer cells were medicated at several concentrations (0, 12.5, 25, 50, 100 μg/mL) for 24 h. In the cytotoxicity experiment, we used MTT solution for measuring the cell toxic level. The cytotoxicity in cancer-free RAW 264.7 cells was evaluated, and samples were determined to be safe. It was observed that the cell viability of RAW 264.7 cells showed low toxicity with G-Rh1 at 100 μg/mL after 24 h (Figure 9A).

However, 100 μg/mL of G-Rh1 significantly inhibited around 40% cell proliferation in the A549 cells compared with the positive control such as commercial cisplatin, which is used as an anticancer drug (Figure 9B). In contrast, it was observed that G-Rh1 decreased A549 cell viability in a dose-dependent manner. Meanwhile, at 100 µM, Rh1 decreased approximately 30% of the cell viability of A549 cells [62] and approximately 25% of the cell viability of HeLa cells [63]. These findings demonstrate that Rh1 promotes the apoptotic pathway in A549 lung cancer cells, which has anti-cancer properties.

### 2.5. In Vitro ROS Induced by G-Rh1

**Figure 10:**
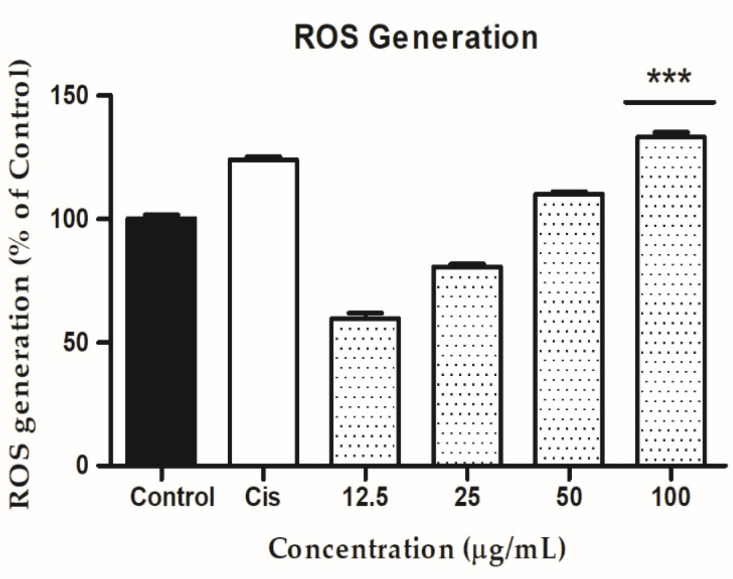
In A549 cells, the ability of ginsenoside Rh1 to generate intercellular reactive oxygen species (ROS) was compared to a positive control (cisplatin). Graph shows mean ± SD values of three replicates. *** *p* < 0.001 indicates significant differences from control groups.

In cancer cells, reactive oxygen species (ROS) play a key role in generating cytotoxicity. In a variety of human cancer cell lines, higher accumulation of ROS level has been shown to cause apoptosis, autophagy, and cell cycle arrest [64,65]. G-Rh1 are important reputed materials for anticancer activity due to reactive oxygen species (ROS) generation [48]. Herein, Intracellular ROS level was determined by the DCFH-DA reagent with cisplatin, G- Rh1 on A549 cells. The G-Rh1 treatment of A549 malignant cells revealed a dose-dependent increased in intracellular ROS generation at higher concentrations (100 μg/mL) in compared to the positive control drug cisplatin (Figure 10). Several studies introduced that mitochondria produce reactive oxygen species (ROS) during apoptosis, and a reduction in mitochondria membrane potential results in increased ROS production and apoptosis [66,67]. In addition, the accumulating ROS can cause p53 to be expressed, which has significant impact on the beginning of apoptosis via transactivating pro-apoptotic proteins (Bax) or interacting with anti-apoptotic mitochondrial proteins (Bcl-2) [68,69]. The inhibition of cell growth and creation of ROS by G-Rh1 extracts in A549 lung cancer cells suggest that ROS production causes apoptosis via the mitochondrial route.

### 2.6. G-Rh1 Induced Apoptosis by Regulating Apoptotic Gene Expression

**Figure 11:**
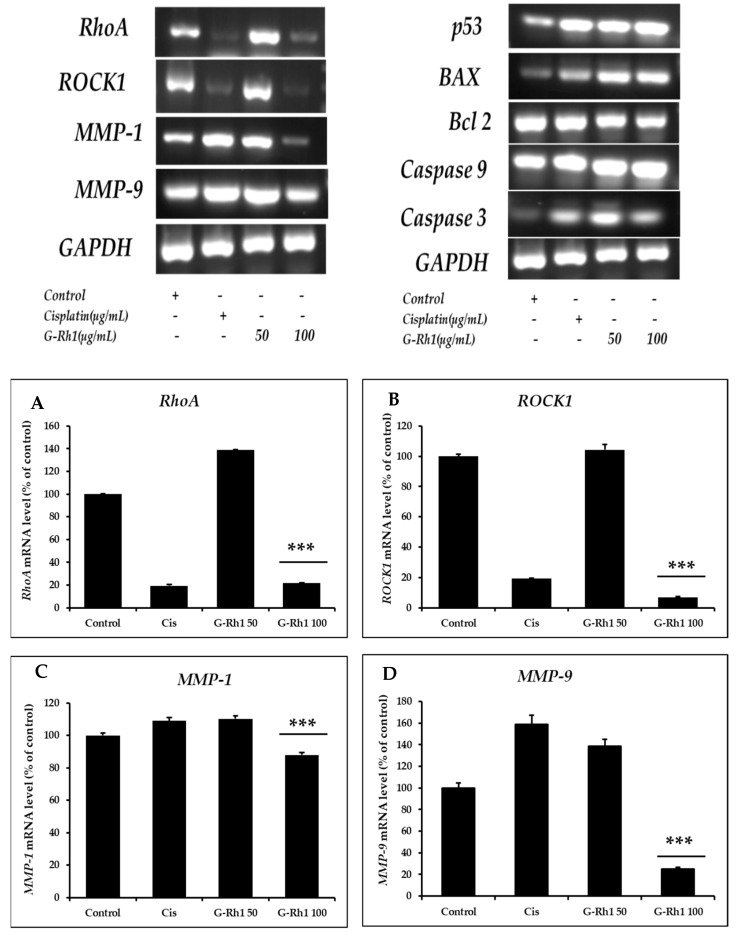

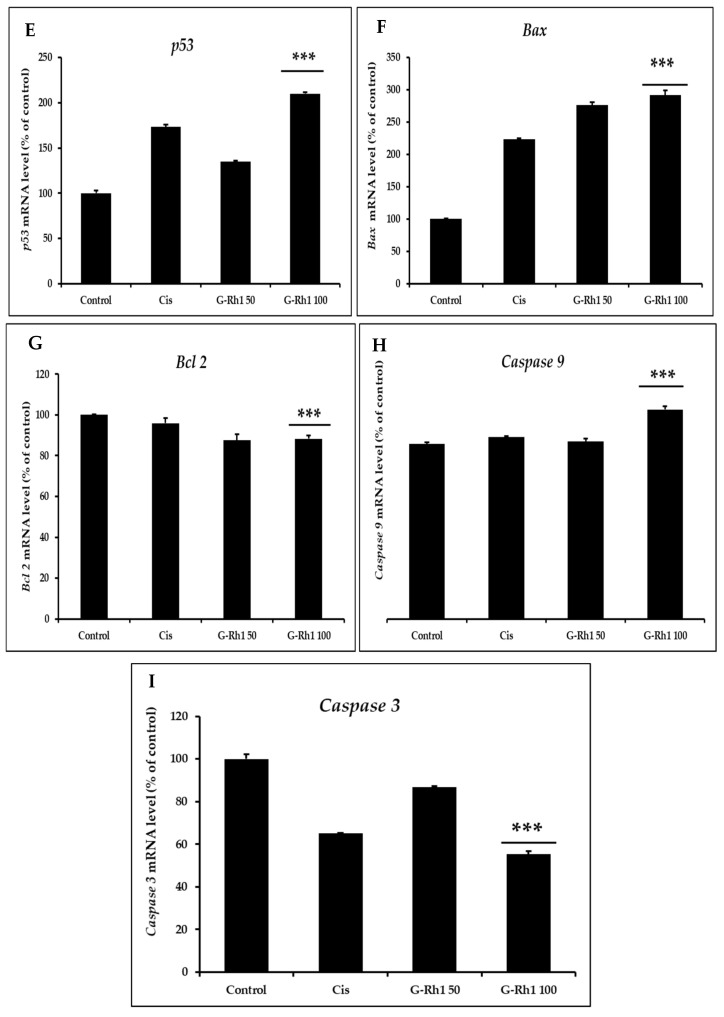
Effects of G-Rh1 on mRNA expression levels of apoptosis-related genes in A549 cells. Here, A549 cells were treated with G-Rh1 at 50 and 100 μg/mL for 24 hr. Subsequently, total RNAs were extracted, and the mRNA expression levels were determined by RT-PCR analysis with different apoptotic genes including (**A**) ROCK, (**B**) RhoA, (**C**) MMP-1, (**D**) MMP- 9, (**E**) p53, (**F**) Bax, (**G**) Bcl2, (**H**) Caspase 9, (**I**) Caspase 3 and compared with those of GAPDH. In A549 cells, RhoA, ROCK1, MMP1, and MMP9 mRNA expression was decreased by G-Rh1. While downregulating Bcl2, G-Rh1 increased p53, Bax, Caspase 3, and Caspase 9. The data shown are representative of the mean values of three independent experiments ±SD. *** *p* < 0.001 as compared to the non-treated control.

To further explore the mechanism involved in the ginsenoside Rh1-mediated anti-lung cancer effect, several essential anti-lung cancer genes were evaluated by RT-PCR using the primers listed in (Section 3.10), including *RhoA, Rock1, MMP1*, *MMP9*, *Bax*, Bcl2, p53, Caspase 3, Caspase 9. Additionally, RhoA and ROCK 1/2 are critical regulators of focal adhesion, actomyosin contraction, proliferation, apoptosis, and cell motility [70,71]. The role of the Rho/ROCK1 signaling pathway in the molecular migration and invasion process has been studied [72,73]. The ROCK pathway is frequently elevated in NSCLC and is related to a more aggressive phenotype. A previous study revealed that in NSCLC, the Nrf2/Keap1 pathway affects cell motility by dysregulating the RhoA/ROCK1 signaling pathway [9]. The treatment with baicalein on A549 delayed the ability to form vasculogenic mimicry and decreased tumorigenicity. These findings were accompanied by downregulated RhoA/ROCK proteins and compromised F-actin cytoskeleton in vivo and in vitro studies [74]. Moreover, the results showed that Rho A and ROCK1 are significantly downregulated on A549 treatment dose-dependent manner by the G-Rh1 at 100 µg/mL (Figure 11).

Matrix metalloproteinases (MMPs), including MMP-1 and MMP-9, are a class of zinc-dependent metalloenzymes that control several physiological functions, including the proliferation and metastasis of tumor cells [75,76]. Therefore, we investigated the mRNA expression level of the MMP-1 and MMP- 9 for anti-cancer activity on A549 cells. Previous research has discovered that G-Rh1 suppresses MMP-1 expression by inhibiting AP-1 and MAPK signaling pathways in human hepatocellular carcinoma cells [49]. Additionally, the decrease in MMP-9 mRNA level caused by DNAzyme inhibited cell migration, proliferation, and invasion in A549 cells [77]. Therefore, in the current study, MMP1 and MMP9 expression levels were examined and shown to be lower after Rh1 treatment on A549 cells.

The generation of ROS by mitochondria is necessary for redox signaling, whereas p53 is a redox-active transcription factor that suppresses cancers. As a result, ROS causes apoptosis in cancer cells via activating p53 [78,79]. p53 regulates the expression of several pro- and anti-apoptotic genes, including Bax and bcl2. Bcl2 is one of the apoptosis mechanisms, that is triggered by stressful situations, including cytokine deficiency or DNA damage [80]. This process may be happened by the activation of mitochondrial-mediated apoptosis, which is indicated by the inhibition of bcl2, an increase in Bax, permitting the release of cytochrome C into the cytoplasm, and ultimately the upregulation of caspase 9/3 gene [81,82]. Additionally, The RT-PCR (Figure 9) showed upregulation of p53 and bax, caspase 3, caspase 9 and downregulation of bcl2 gene expression in a dose-dependent manner by G-Rh1 compared to commercial drug cisplatin. Additionally, The RT-PCR (Figure 11) showed upregulation of p53 and bax, caspase 3, caspase 9 and downregulation of bcl2 gene expression in a dose-dependent manner by G-Rh1 compared to commercial drug cisplatin. Therefore, this study revealed that G-Rh1 has ability to inhibit the apoptosis gene expression in Lung cancer cells. However, Quantitative analysis and further research into molecular mechanisms are required to understand the biological pathways fully.

## 3. Materials and Methods

### 3.1. Chemical

G-Rh1 samples were collected from the lab of Hanbang bio, Suwon, Korea. The Korean Cell Line Bank provided the lung cancer cell line (A549) and Raw 264.7 murine macrophage cells used in this investigation (KCLB, South Korea). Fetal bovine serum (FBS) and penicillin-streptomycin solution were purchased from Gen DEPOT (Barker, TX, USA). Gibco (Waltham, MA, USA) supplied high glucose, pyruvate Dulbecco’s Modified Eagle’s Medium (DMEM). (3-(4, 5-dimethylthiazol-2-yl)-2,5-diphenyltetrazolium bromide or MTT) the solution from Life Technologies, Suwon, Korea.

### 3.2. Computational Experiments

#### Protein and Ligand Preparation

The most active compound from the *Panax ginseng* medicinal plant, ginsenoside Rh1, was selected for this study. The ginsenoside Rh1 compound structure was collected from our own in-house database (Figure 1A). The two-dimensional structure (2D) of ginsenoside Rh1 was drawn using ChemSketch, http://www.acdlabs.com (accessed on 2 August 2022) (Advanced Chemistry Development, Inc. Toronto, ON, Canada). The drawn 2D structure file was in mol format, which was then converted into a three-dimensional (3D) structure as .pdb format by importing it into Discovery Studio 3.5 visualizer (DS 3.5) (DS, http://www.accelrys.com (accessed on 3 August 2022); Accelrys, Inc. San Diego, CA, USA). The known ROCK1 inhibitor, fasudil (M77), was used as a control ligand for docking simulation and was retrieved from the ROCK1(PDB ID: 5WNE) crystal complex structure. The known RhoA inhibitors, GDP (control from the PDB complex of RhoA (PDB ID: 4D0N), fasudil, Ibuprofen [83], and Roshin [84] were used as control. Furthermore, these molecules were optimized using the Conjugate Gradients method [54] followed by Steepest Descent [85] in 200 steps using the PyRx program [86]. The minimization step was carried out using Universal Force Field (UFF) [87].

### 3.3. Pharmacokinetic Properties Prediction

#### ADMET and Drug Likeness Prediction

Based on the chemical structure, predicting the pharmacokinetic properties of a molecule is an essential task in drug design. Descriptors such as physiochemical and pharmacokinetic properties were calculated using the Qikprop version 3.0 module encoded by the Schrödinger program (http://www.schrodinger.com (accessed on 5 June 2022)). These properties, called ADMET (absorption, distribution, metabolism, excretion, and toxicity), are essential for identifying active saponins. Ginsenoside Rh1 was imported and neutralized using Maestro GUI wizard before calculating physiochemical and pharmacokinetic property prediction because we want to predict the properties of interest at neutral pH and not when the molecule is at an ionized state. This step is essential because neutralized ginsenoside Rh1 can generate pharmacokinetic properties. Furthermore, Qikprop provides a range for comparing a particular molecule’s properties with those of 95% of the known drugs. The hepatotoxicity and CYP2D6 inhibition scores were calculated using the ADMET module available from the DS 3.5 program. The following ADMET descriptors were computed for ginsenoside Rh1: aqueous solubility, serum protein binding, logP for octanol/water, hepatotoxicity, CYP2D6 inhibition probability, and human oral absorption in GI (%). Further, drug-likeness screening was carried out by Lipinski’s rule of five [88]. This filtration is mainly used for eliminating non-drug-like molecules and selecting drug-like molecules based on Lipinski’s rules: molecular weight (MW), LogP, the number of hydrogen bond donors (HBD), and the number of hydrogen bond acceptors (HBA). These properties can be determined drug-likeness property of ginsenoside Rh1.

### 3.4. In Silico Biological Activity, Target and Active Site Prediction

Another computational program called Prediction of Activity Spectra for Substances (PASS) (http://www.pharmaexpert.ru/passonline/ (accessed on 3 April 2022)) was used to predict the biological activity spectrum based on the chemical structure formula [89]. This computational technique was used to predict what type of biological activities of ginsenoside Rh1 are present in the biological system. In addition, this method produces a list of biological activity along with the probability of active (Pa) and probability of inactive (Pi) values. The accuracy of biological activity was carefully measured based on the predicted probability scores; when Pa is close to 1 and Pi close to zero, the molecule has a higher probability of biological activity. In addition, ginsenoside Rh1 has been validated using the SWISS target prediction tool (Figure S5), which aims to predict the most probable protein targets of small molecules [90].

We have applied the DoGSiteScorer online tool to predict binding pockets within native ROCK1 and RhoA (Table S1 and Figure S6). DoGSiteScorer is a grid-based method that uses a Difference of Gaussian filter to detect potential binding pockets solely based on the 3D structure of the protein and splits them into subpackets. Global properties are calculated, describing the size, shape, and chemical features of the predicted (sub) pockets. Per default, a simple draggability score is provided for each (sub) pocket based on a linear combination of the three descriptors describing volume, surface, hydrophobicity, and enclosure. The binding pockets are ranked according to their size, surface area, and draggability score [91].

### 3.5. Molecular Docking

The ROCK1 protein [PDB ID: 5WNE] and RhoA protein [PDB ID: 4D0N] 3D structure was retrieved from the Protein Data Bank (PDB) [92,93] along with the co-crystalized ligand of fasudil and GDP. To perform the docking studies, the ROCK and RhoA structure was prepared by Autodock tool graphical interface (GUI) [94]. A known inhibitor fasudil and GDP along with the water molecules were removed from the original structure and made as a free receptor [58]. Further, Kollman charges and polar hydrogens were added to the receptors. The fasudil and GDP binding sites are the most likely active sites. Further, fasudil is involved in two hydrogen bond interactions with the M156 and D160 residues and other studies have shown that these residues play a role in ROCK1 inhibition [95].

The energy-minimized ginsenoside Rh1 with ROCK1 and RhoA protein structures were used to perform docking simulations by the Autodock Vina program [89]. The fasudil and GDP compound was used as a control for this study. The detailed docking procedures were followed according to our previous study [96,97]. The potential binding interaction was identified based on binding affinity scores and hydrogen bond interactions between ROCK1 and RhoA with ginsenoside Rh1. The results of each complex were saved from the graphical interface of Autodock tools and imported to DS.3.5 visualizer to analyze their interactions at the molecular level.

### 3.6. Molecular Dynamics Simulations

To check the stability of ginsenoside Rh1 along with ROCK1 and RhoA protein, it was subjected to a molecular dynamics (MD) study using the Gromacs 4.6 (GROningen MAchine for Chemical Simulations) program [98]. The ROCK1 protein (apo form), ROCK1-fasudil (known inhibitor), ROCK1-dexamethasone (control drug), ROCK1-ginsenoside rh1, RhoA protein (unbound), RhoA-GDP (inhibitor), RhoA-dexamethasone (control drug), and RhoA- ginsenoside rh1 complex structures were used to perform MD simulations. The ROCK1 and RhoA protein topology files were created using the gromacs utility (pdb2gmx), and ginsenoside Rh1 topology files were created using the Dundee PRODRG2 server [99]. For all simulations, we used the single-point-charge (SPC) [55] water model and the Gromacs [100] force field. The detailed MD simulation procedure was followed according to our previous study [96,101]. Each system (ROCK1, ROCK1-fasudil, ROCK1-dexamethasone, ROCK1-ginsenoside rh1, RhoA protein, RhoA-GDP, RhoA-dexamethasone, and RhoA- ginsenoside rh1) was neutralized by adding the appropriate ions (Na+ or Cl-) and canonical (NVT) ensemble, and isothermal-isobaric (NPT) ensemble equilibration steps were performed. Further, 50 nano-second (ns) production was carried out individually for all systems. To analyze the MD simulation of ROCK1-ginsenoside Rh1, the results were saved every 2 pico seconds (ps). In addition, the root means square deviation (RMSD), root mean square fluctuation (RMSF), H-Bonds, and secondary structures were computed using gromacs utilities such as g_rms, g_rmsf, and g_hbond. The docking and molecular dynamics simulations were performed using an Intel^®^ 2.93 GhZ Xenon^®^ CPU 5670 CentOS server. Lastly, we have employed the gmx_MMPBSA [102] package for free energy calculations based on the single trajectory of GROMACS with an appropriate force field. This tool allows free energy calculations using MM/PBSA or GBSA (Molecular Mechanics/Poisson-Boltzmann or Generalized Born Surface Area) methods with an implicit solvent model.

### 3.7. Principal Component Analysis

To determine the ROCK1 protein structure change upon ligand binding, we used gromacs utilities. A PCA method called essential dynamics (ED) was followed according to this protocol [103]. The protein coordinates of ROCK1 and its complexes (ROCK1-fasudil and ROCK1-ginsenoside Rh1) were used as a starting point to calculate the PCA using g covar gromacs utilities. This program computes the corresponding protein motions of eigenvectors, eigenvalues, and projection values. In addition, these principal components were determined based on the mass-weighted covariance matrix of the backbone atoms of each structure. Finally, the g_anaeig gromacs utilities were used to analyze the protein motions based on the 2D projection values for the first two principal components. All dynamics graphics were plotted using Microsoft Excel and the XMGRACE [104] program.

### 3.8. Cell Culture

Human lung cancer (A549) was grown in a medium containing 89 percent RPMI 1640, 10% FBS, and 1% penicillin-streptomycin. RAW 264.7 murine macrophage cells were generally purified in DMEM with 10% FBS and 1% penicillin-streptomycin. Two cell lines were allowed to adhere and grow for 24 h before being treated with different samples in a 37 °C humidified incubator with a 5% CO_2_ environment.

### 3.9. In Vitro Cytotoxicity of Ginsenosides Rh1

Using an MTT solution, the cytotoxicity of G-Rh1 was investigated in A549 and RAW 264.7 cell lines. The cytotoxicity of cisplatin (10 μg/mL) was evaluated on just A549 cells, and the findings were compared to G-Rh1 after 24 h. The cell viability assay was carried out as previously stated [105]. In a 96-well plate, cancer cells and normal cells were first plated at a selective density of 1 × 10^4^ cells/well. After that, cells were treated with a variety of concentrations (0, 12.5, 25, 50, 100 μg/mL) and left to incubate for 24 h. After 24 h, cells were treated for 3–4 h at 37 °C with 20 μL of 3-(4, 5-dimethyl-2-thiazolyl)-2, 5- diphenyl tetrazolium bromide solution (MTT; 5 mg/mL, in PBS; Life Technologies, Eu- gene, OR, USA). Furthermore, the addition of MTT reagents results in the formation of purple-colored formazan in live cells. For dissolving the insoluble formazan agents, 100 μL of DMSO was added to each well. The data were acquired using a 570 nm ELISA reader (BioTek Instruments, Inc., Winooski, VT, USA).

### 3.10. Reactive Oxygen Species (ROS) Assay

In human lung cancer (A549), 2′,7′dichlorodihydrofluorescein diacetate (DCFH-DA) was used to measure the strength of reactive oxygen species (ROS). To allow attachment in 96-well cell culture plates, we seeded the cells at a density of 1 × 10^4^ per well and left them in the incubator overnight for 100 percent growth confluency. A549 cells were then treated for 24 h with various concentrations of cisplatin (10 μg/mL), (0, 12.5, 25, 50, 100 μg/mL). After 24 h of treatment, the cells were stained with 100 μL of DCFH-DA (10 μM) solution in each well and incubated for 30 min in the dark. The cells were then washed twice with PBS ((100 μL/well), and the old medium was discarded. At an excitation wavelength of 485 nm and an emission wavelength of 528 nm, a multi-model plate reader (spectrofluorometer) was utilized to determine the fluorescence intensity of ROS production. DCFH-DA reagent was used to measure the increase in ROS.

### 3.11. Reverse Transcription Polymerase Chain Reaction (RT-PCR)

Total RNA from A549 cells was obtained using QIAzol lysis reagent (QIAGEN, Germantown, MD, USA), and the reverse transcription process was carried out using 1μg of RNA in 20 μL of amfiRivert reverse transcription reagents (GenDepot, Barker, TX, USA), as directed by the manufacturer. The obtained cDNA was amplified with the following primers: RhoA, forward: 5′-CAG CAA GGA CCA GTT CCC AGA-3′ and Reverse: 5′-TGC CAT ATC TCT GCC TTC TTC AGG-3′, ROCK1, forward: 5′-AGG AAG GCG GAC ATA TTA GTC CCT-3′ and reverse: 5′-AGA CGA TAGTTGGGTCCCGGC-3′, MMP-1, forward: 5′-ATT CTA CTG ATA TCG GGG CTT TGA -3′ and reverse: 5′-ATG TCC TTG GGG TAT CCG TGT AG-3′, MMP-9, forward: 5′-CGT CGT GAT CCC CAC TTA CT-3′ and MMP-1, forward: 5′-ATT CTA CTG ATA TCG GGG CTT TGA -3′ and reverse: 5′-AGA GTA CTG CTT GCC CAG GA -3′, BAX, forward 5′-GGT TGC CCT CTT CTA CTT T-3′ and reverse 5′-AGC CAC CCT GGT CTT G-3′; bcl2, forward 5′-GAA GGG CAG CCG TTA GGAAA-3′ and reverse 5′-GCG CCC AAT ACG ACC AAA TC-3′; p53, forward 5′-TCT TGGGCC TGT GTT ATC TCC-3′ and reverse 5′-CGC CCA TGC AGG AAC TGT TA-3′, CASPASE 3, forward 5′-GAA GGA ACA CGC CAG GAA AC-3′ and reverse 5′-GCA AAG TGA AAT GTA GCA CCA A-3′; CASPASE 9, forward 5′-GCC CGA GTT TGA GAG GAA AA -3′ and reverse 5′-CAC AGC CAG ACC AGG AC -3′; and GAPDH, forward 5′-CAA GGT CAT CCA TGA CAA CTT TG-3′ and reverse 5′-GTC CAC CAC CCT GTT GCT GTA G-3′. The reaction was repeated 35 times for 30 s at 95 °C, 30 s at 60 °C, and 50 s at 72 °C. The amplified RTPCR results were examined on 1% agarose gels, stained with Safe Pinky DNA Gel Staining (GenDepot, Barker, TX, USA), and captured under UV light.

## 4. Conclusions

In this study, the well-known compound G-Rh1 from *Panax ginseng* Meyer was validated for its potential to inhibit RhoA and ROCK1 (metastasis) and to regulate RhoA/rock/p53/MMP-1/MMP-9 (apoptosis). Molecular docking, biological activity prediction, molecular dynamics modeling, MM-PBSA, principal component analysis, and ADMET prediction were employed to determine the biological activity of G-Rh1. Further, in vitro validations were conducted to observe the anti-lung cancer activity of G-Rh1 on human lung cancer cells (A549). As a result, the pharmacokinetic findings reveal that G-Rh1 has the potential to be a drug-like molecule. Additionally, when G-Rh1 interacts with the residues in the ROCK1 active site, strong hydrogen bonds and hydrophobic interactions are formed that distinctly outline the inhibitory effect of RhoA and ROCK1. In vitro cytotoxicity and ROS studies disclose that G-Rh1 was not toxic to non-cancerous RAW 264.7 cells at concentrations up to 100 μg/mL. In contrast, G-Rh1 showed more toxicity on cancerous cells at up to 100 μg/mL. Furthermore, G-Rh1 induced higher ROS levels in human lung carcinoma cells at higher concentrations. Moreover, G-Rh1 reduced the RhoA and ROCK1 gene expression in the A549 lung cancer cells. On the other hand, G-Rh1 regulated the gene expression of apoptosis regulators such as p53, Bax, Bcl2 Caspase 3, Caspase 9. According to the current findings, ROCK1 and RhoA downregulation inhibited NSCLC A549 cell motility, proliferation, and survival. Hence, the proposed G-Rh1 compound should be subjected to further experimental validation and may be used as a lead molecule for ROCK1 and RhoA inhibition and apoptosis regulation in treating and managing lung cancer.

## Figures and Tables

**Table 1:** ADMET results of ginsenoside Rh1 with pharmacokinetic properties.

QP (%)	LogS	QplogKhsa	CYP2D6 Inhibition	Hepato Toxicity	QPlogPo/w	Reference
49.4	−4.829	0.1	0.2	0	2.6	[54]

QP (%): Percentage of human oral absorption in GI (acceptable range: <25% is poor and >80% is high). LogS: Aqueous solubility (acceptable range: −6.5 to 0.5). QPlogKhsa: Serum protein binding (acceptable range: −1.5 to 1.5). CYP2D6 inhibition: 0 is non-inhibitor and 1 is inhibitor. Hepatotoxicity: 0 is non-toxic and 1 is toxic. QPlogPo/w: Octanol/water partition coefficient (acceptable range −0.2 to 6.5).

**Table 2:** Drug-likeness prediction results of ginsenoside rh1 based on Lipinski rule of 5.

Properties	Predicted Values
Molecular weight	638.8
Hydrogen bond donor	7
Hydrogen bond acceptor	14
LogP	2.625

**Table 3:** Predicted biological activity of ginsenoside Rh1.

Predicted Biological Activity	Pa ^a^ (%)	Pi ^b^ (%)
Chemopreventive	0.99	0.001
Caspase 3 stimulant	0.99	0.001
Dementia treatment	0.98	0.000
Vascular dementia treatment	0.98	0.000
Antithrombotic	0.98	0.001
CYP3A inducer	0.97	0.001
CYP3A4 inducer	0.96	0.001
Apoptosis antagonist	0.96	0.001
Hepatoprotectant	0.94	0.002
CYP2C9 inducer	0.93	0.001

^a^ Pa represents the probability of active. ^b^ Pi represents the probability of inactive.

**Table 4:** Interaction of ginsenoside Rh1 and control drugs with amino acid residue of ROCK1 & RhoA.

Protein	Compound	Binding Energy (kcal/mol)	Hydrogen Bond Interactions	Hydrophobic Interactions	No. of Hydrogen Bonds
**ROCK1**	Ginsenoside Rh1	−8.9	ALA86, ASP160, ASN203, ASP216	GLY85, ILE82, VAL162	3
	Dexamethasone	−8.4	ALA86, ASP216	GLY85, GLY218	2
	Fausidil	−8.3	ARG84, MET156	GLY83, VAL90, ALA103, ALA215, LEU205	2
**RhoA**	Ginsenoside Rh1	−7.1	ARG5, ASP78, PRO180, GLN180	ASP78, LYS6, PRO75, PHE106	4
	Dexamethasone	−7.0	ASP76, ASP78, ALA177	ALA181, THR77, PRO75, PRO108	3
	GDP	−7.2	ALA15, CYS16, GLY17, THR19, CYS20, ALA161, LYS162	LYS18, LYS118	7
	Fausidil	−5.5	GLU32, TYR34, VAL33	PRO31, LYS27, PRO36	3
	Ibuprofen	−6.3	GLU40, VAL38	LYS27	2
	Rhosin	−7.1	GLU40, VAL38	PRO36, LYS27, TYR42	2

**Table 5:** Calculated binding free energy (MM-PBSA).

Complex	ROCK1-Ginsenoside Rh1 Complex	ROCK1-Dexamethasone Complex	ROCK1-Fausidil Complex
**ΔVDWAALS (kcal/mol)**	−39.8	−29.79	−34.47
**ΔEEL (kcal/mol)**	−20.78	−18.77	−18.09
**ΔEPB (kcal/mol)**	34.6	36.09	38.88
**ΔENPOLAR (kcal/mol)**	−24.80	−21.56	−23.91
**ΔEDISPER (kcal/mol)**	42.9	42.97	43.63
**ΔGGAS (kcal/mol)**	−50.6	−48.57	−52.6
**ΔGSOLV (kcal/mol)**	61.8	57.51	59.6
**ΔTOTAL (kcal/mol)**	−30.80	−20.95	−27.04

## Data Availability

The concern of corresponding author data can be provided.

## Supplementary Materials

The following supporting information can be downloaded at: https://www.mdpi.com/1420-3049/27/23/8311/s1, Figure S1: The secondary structural elements of (A) ROCK1 protein, (B) ROCK1-fasudil, and (C) ROCK1-ginsenosde Rh1 during the 10 ns simulation. The color encoded is based on the DSSP program in gromacs utilities; Figure S2: Docking interactions of control drug inhibitors ((A) Dexamethasone, (B) Fausidil with ROCK1); Figure S3: Docking interactions of control drug inhibitors ((A) Dexamethasone, (B) GDP, (C) Fausidil, (D) Ibuprofen, (E) Rhosin) with RhoA); Figure S4: The different conformation snapshots of (A) ROCK1 protein, and (B) ROCK1-ginsenosde Rh1 through a 10 ns simulation; Figure S5: Validation of target prediction for G-Rh1 using SWISS target prediction; Figure S6: (A) Predicted active site for ROCK1 (B) Predicted active site for RhoA. Table S1. Active site prediction for ROCK1 and RhoA using DoGSiteScorer.
