# Supplementary material for: Investigating the Anticancer Activity of G-Rh1 Using In Silico and In Vitro Studies (A549 Lung Cancer Cells)
Source: Molecules. 2022 Nov 28;27(23):8311. doi: 10.3390/molecules27238311 (PMC9890317; doi:10.3390/molecules27238311)
Supplement: Supplementary file 1 [file molecules-27-08311-s001.zip › molecules-1979831-Supplementary Figure List.pdf]

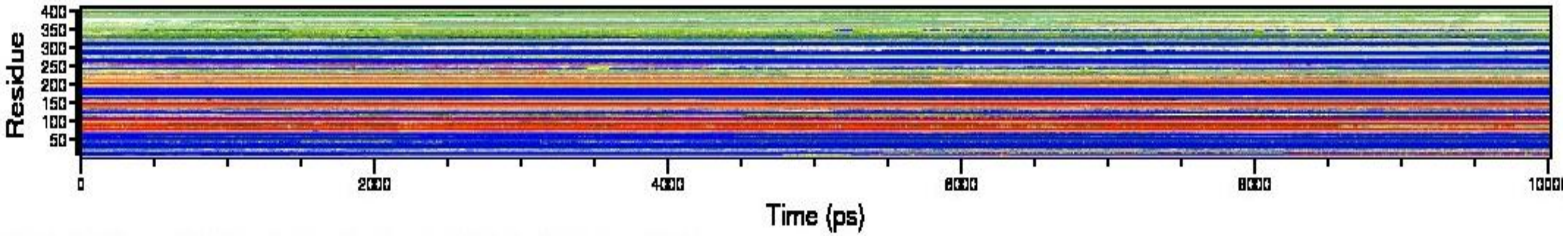

(a)

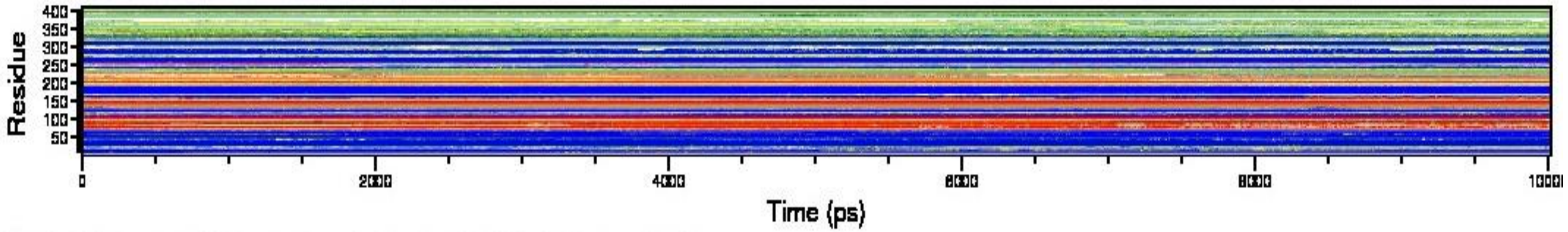

(b)

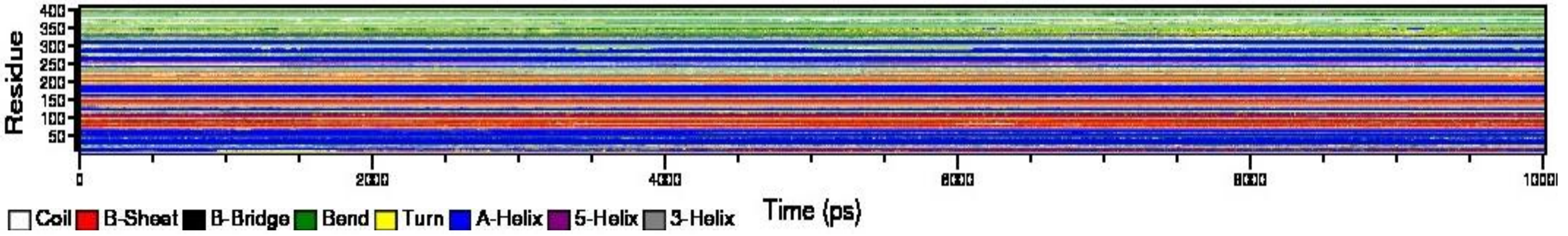

(c)

Fig. S1: The secondary structural elements of (A) ROCK1 protein, (B) ROCK1-fasudil, and (C) ROCK1-ginsenosde Rh1 during the 10 ns simulation. The color encoded is based on the DSSP program in gromacs utilities.

(A) Dexamethasone - ROCK1 Protein **(B)** Fausidil - ROCK1 Protein

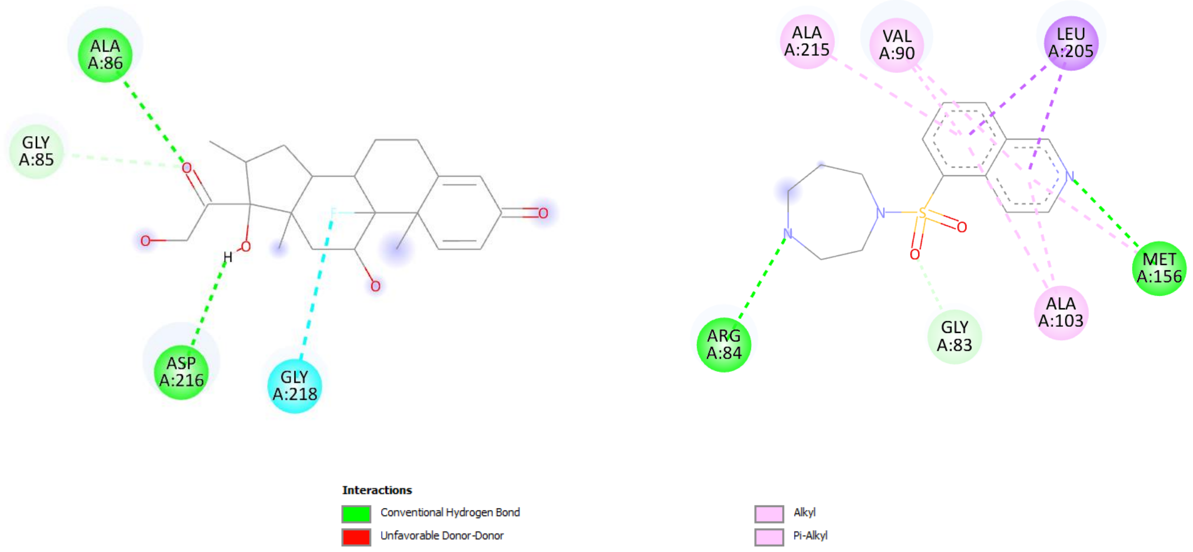

Fig. S2. Docking interactions of control drug inhibitors (**(A)** Dexamethasone, **(B)** Fausidil with ROCK1).

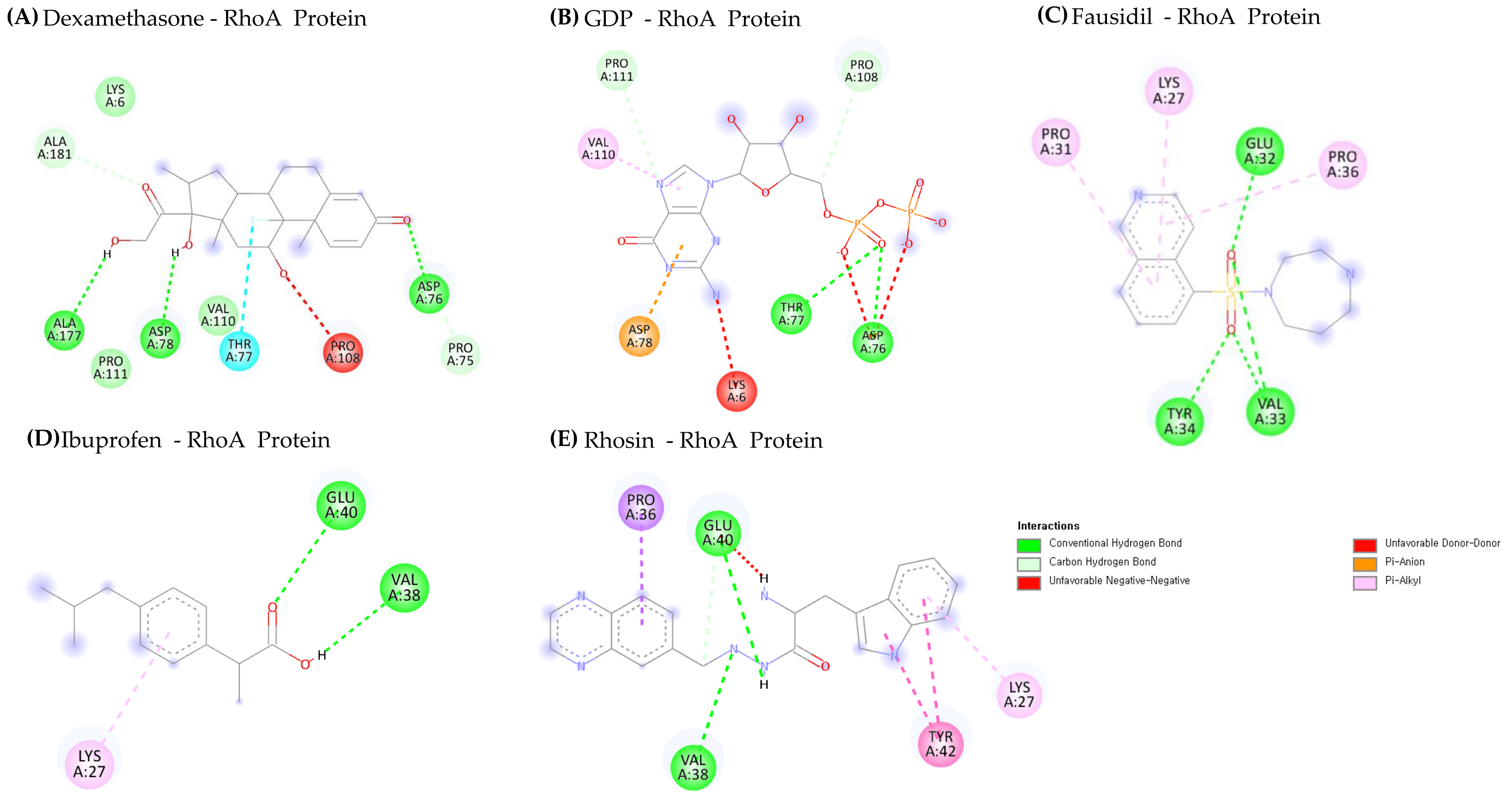

Fig. S3. Docking interactions of control drug inhibitors (**(A)** Dexamethasone, **(B)** GDP, (C) Fausidil, (D) Ibuprofen, (E) Rhosin) with RhoA).

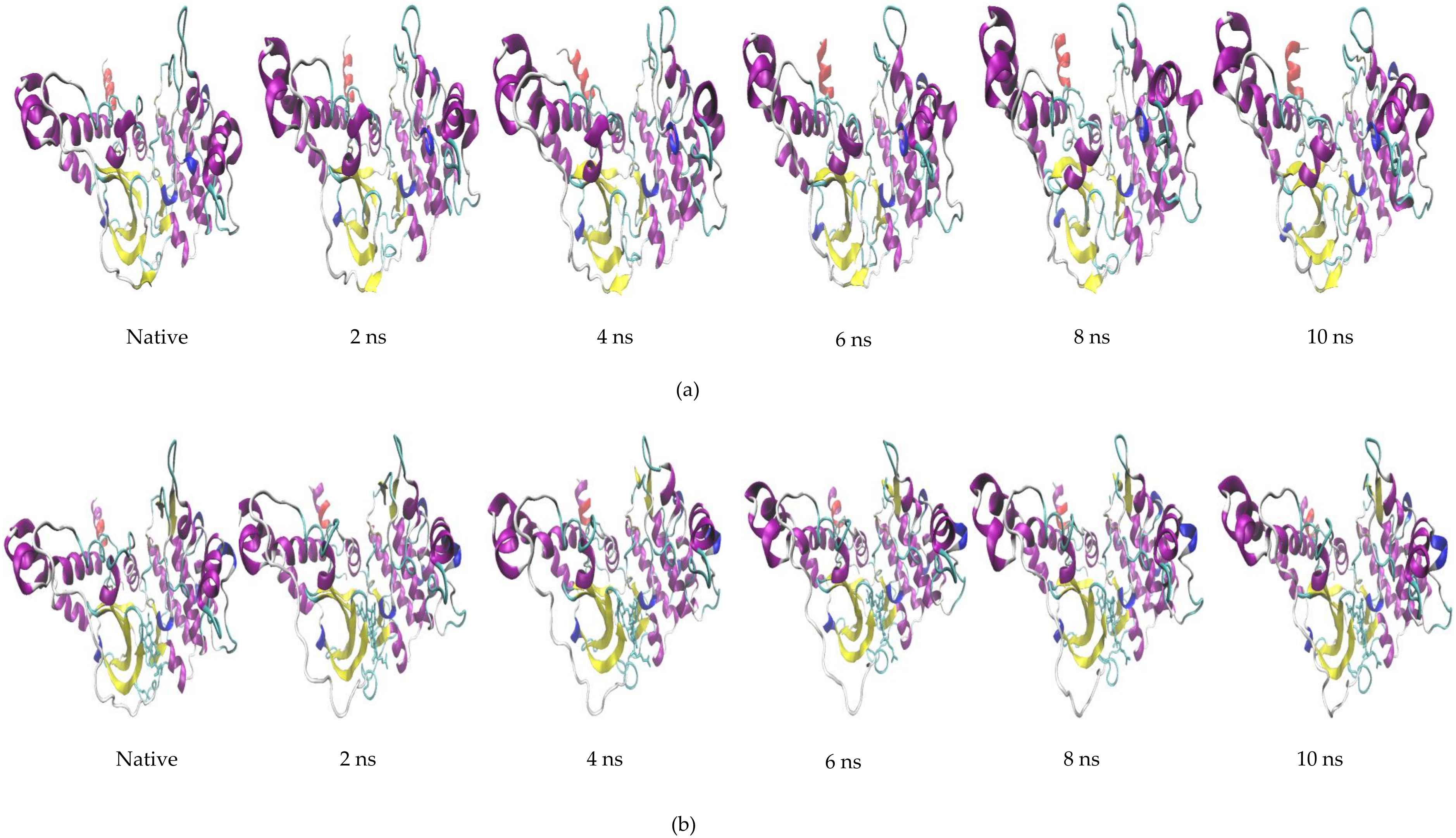

Fig. S4: The different conformation snapshots of (A) ROCK1 protein, and (B) ROCK1-ginsenosde Rh1 through a 10 ns simulation.

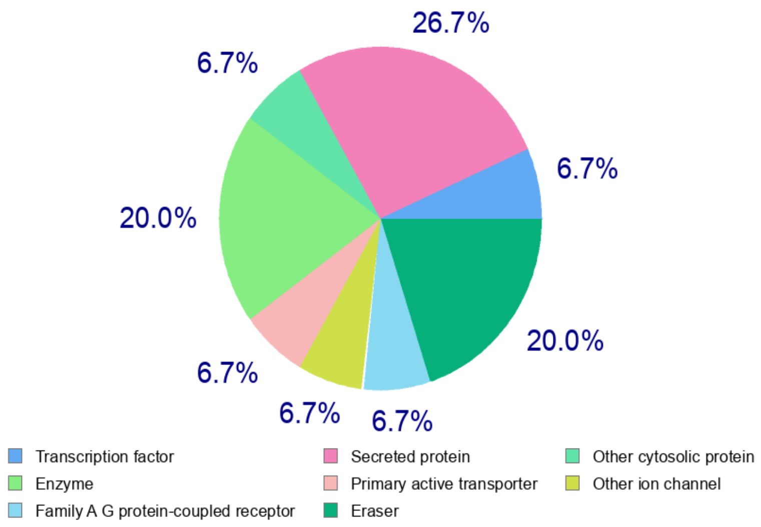

Target	Common name	Uniprot ID	ChEMBL ID	Target Class	Probability*
Apoptosis regulator Bcl-X	BCL2L1	Q07817	CHEMBL4625	Other ion channel	
Bcl-2-related protein A1 *(by homology)*	BCL2A1	Q16548	CHEMBL6044	Unclassified protein	
Matrix metalloproteinase 2	MMP2	P08253	CHEMBL333	Protease	
Matrix metalloproteinase 8	MMP8	P22894	CHEMBL4588	Protease	
Matrix metalloproteinase 13	MMP13	P45452	CHEMBL280	Protease	
Matrix metalloproteinase 1	MMP1	P03956	CHEMBL332	Protease	
Matrix metalloproteinase 14	MMP14	P50281	CHEMBL3869	Protease	

Fig. S5: Validation of target prediction for G-Rh1 using SWISS target prediction.

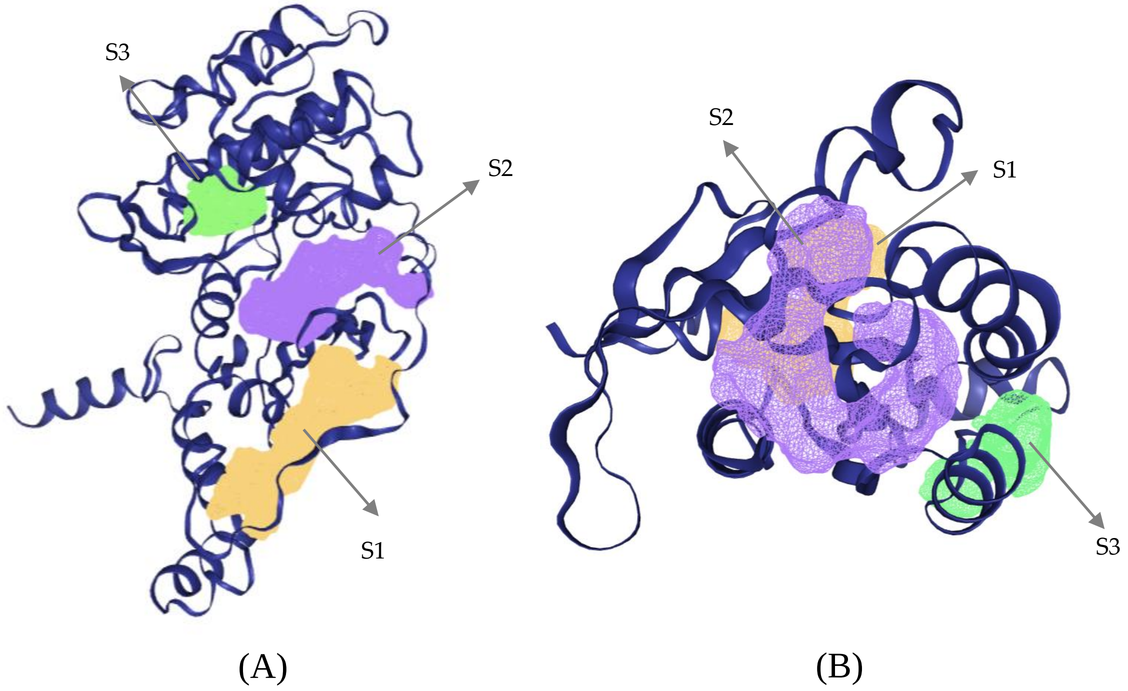

Fig. S6: (A) Predicted active site for ROCK1 (B) Predicted active site for RhoA.
