# Supplementary material for: Investigating the Anticancer Activity of G-Rh1 Using In Silico and In Vitro Studies (A549 Lung Cancer Cells)
Source: Molecules. 2022 Nov 28;27(23):8311. doi: 10.3390/molecules27238311 (PMC9890317; doi:10.3390/molecules27238311)
Supplement: Supplementary file 1 [file molecules-27-08311-s001.zip › molecules-1979831-Supplementary table List.pdf]

Table S1. Active site prediction for ROCK1 and RhoA using DoGSiteScorer.

Protein	Site	Volume Å^3	Surface Å^2	Drug Score	Simple Score
ROCK1	S1	804.16	1173.88	0.86	0.49
	S2	799.49	975.71	0.84	0.54
	S3	163.33	118.09	0.35	0.0
RhoA	S1	540.35	677.26	0.74	0.37
	S2	435.78	737.02	0.65	0.26
	S3	238.78	513.84	0.41	0.04
